# Dose-Related Effects of Alcohol on Cognitive Functioning

**DOI:** 10.1371/journal.pone.0050977

**Published:** 2012-11-29

**Authors:** Matthew J. Dry, Nicholas R. Burns, Ted Nettelbeck, Aaron L. Farquharson, Jason M. White

**Affiliations:** 1 School of Psychology, University of Adelaide, Adelaide, South Australia, Australia; 2 School of Pharmacy and Medical Sciences, University of South Australia, Adelaide, South Australia, Australia; California Pacific Medicial Center Research Institute, United States of America

## Abstract

We assessed the suitability of six applied tests of cognitive functioning to provide a single marker for dose-related alcohol intoxication. Numerous studies have demonstrated that alcohol has a deleterious effect on specific areas of cognitive processing but few have compared the effects of alcohol across a wide range of different cognitive processes. Adult participants (N = 56, 32 males, 24 females aged 18–45 years) were randomized to control or alcohol treatments within a mixed design experiment involving multiple-dosages at approximately one hour intervals (attained mean blood alcohol concentrations (BACs) of 0.00, 0.048, 0.082 and 0.10%), employing a battery of six psychometric tests; the Useful Field of View test (UFOV; processing speed together with directed attention); the Self-Ordered Pointing Task (SOPT; working memory); Inspection Time (IT; speed of processing independent from motor responding); the Traveling Salesperson Problem (TSP; strategic optimization); the Sustained Attention to Response Task (SART; vigilance, response inhibition and psychomotor function); and the Trail-Making Test (TMT; cognitive flexibility and psychomotor function). Results demonstrated that impairment is not uniform across different domains of cognitive processing and that both the size of the alcohol effect and the magnitude of effect change across different dose levels are quantitatively different for different cognitive processes. Only IT met the criteria for a marker for wide-spread application: reliable dose-related decline in a basic process as a function of rising BAC level and easy to use non-invasive task properties.

## Introduction

Alcohol is a general CNS depressant that affects neurological functioning in a dose-dependent manner. The effects of alcohol are mediated through a number of target sites in the brain, principally GABA_A_ and NMDA receptors [1]. At GABA_A_ receptors alcohol acts non-competitively to increase GABA activity. Although there are some differences, this action is shared with other sedatives such as benzodiazepines and barbiturates. At the NMDA receptor alcohol is a non-competitive antagonist, decreasing the activity of the excitatory neurotransmitter glutamate. This combined increase in inhibition and decrease in excitability results in a general CNS depressant effect.

The immediate adverse effects of alcohol on various aspects of cognitive functioning have been well documented [2], [3], [4], [5], [6], [7], [8], [9]. We reviewed articles published between 1990 and the present and found that the majority tended to concentrate upon a single domain of cognitive functioning (or closely related domains). Only a handful of studies have compared the effects of alcohol across a range of cognitive abilities within a single study. Such an approach is potentially highly useful because it permits a direct comparison of the differential effects of alcohol on various aspects of cognitive functioning. Furthermore, it provides the opportunity to investigate changes in the impairment of different cognitive abilities across a range of target blood alcohol concentrations (BACs).

In the present study we compared participants’ performance on six psychometric tests across a range of target BAC levels (0.00, 0.048, 0.082 and 0.10%). Each of the tests have been widely employed as indices of a range of key cognitive processes that have previously been shown to be sensitive to the effects of alcohol intoxication, such as speed of information processing [10], [11], [12], [13], [14], [15], [16], divided attention [17], [18], [19], [20], [21], [22], [23]; problem solving [24], [25], [26], [27], [28], [29], [30], working memory [31], [32], [33], [34], [35], [36], response inhibition and cognitive flexibility [37], [38], [39], [40], [41], [42], and psychomotor functioning [18], [43], [44], [45], [46].

In the following we briefly describe the six tests employed in our study. Further details regarding the tests will be provided (where necessary) in the Method section.

### Inspection Time

[47], [48] is a measure of speed of information processing. Unlike most tests of information processing speed (such as simple- or choice-Reaction Time) IT is motor free; it does not require a speeded response on behalf of a participant, rather it measures the minimum display-time necessary for a participant to make two-alternative forced-choice decision. As noted by Deary et al [49] IT is primarily of interest for three reasons. First, it provides a measure of the lower-bounds of information processing that is sensitive to inter-individual differences; second, it is correlated with higher-level cognitive processes such as those measured by psychometric intelligence tests; and third, it is sensitive to disruptions in brain functioning caused by traumatic injury, degenerative disorders and normal ageing.

### The Traveling Salesperson Problem

[50], [51] is a measure of strategic problem solving. Solving a TSP requires participants to continuously monitor their performance whilst making sequential decisions subject to multiple interacting constraints. The TSP has been widely employed in recent problem solving research [52] and has been shown to be related to measures of general intelligence [53], [54], [55]. Furthermore, the TSP has been shown to be sensitive to age-related differences in cognitive functioning [56] and performance decrements due to neurological dysfunction [57], [58].

### The Useful Field of View test

[59], [60] is a measure of processing speed and divided visual attention. According to Owsley et al [61] task performance is reliant upon both the integrity of the viewer’s visuo-sensory input, as well as higher-level cognitive functions. The test was developed for use in studies on driving and crash risk and has been shown to be predictive of driving ability and everyday functioning in older adults.

### The Self-Ordered Pointing Task

[62] is a measure of working memory function. Performance on the task requires participants to hold visual information in short-term storage while executing a response strategy and continuously monitoring performance. PET scanning indicates that performance on the task is related to activation in areas of the pre-frontal cortex associated with executive functioning [63]. Further, the test has been employed as a test of frontal-lobe dysfunction [64] and cognitive impairment due to normal ageing [65].

### The Sustained Attention to Response task

[66] is a measure of response inhibition and cognitive flexibility. Participants are required to respond quickly to a commonly occurring set of stimuli, but withhold responding to a rarely occurring target stimulus. Fassbender et al [67] demonstrated via fMRI that performance on the task involves regions of the prefrontal cortex associated with inhibitory control, performance monitoring and error processing. Importantly, the task is sensitive to individual differences in attentional failure in both clinical and non-clinical populations [68].

### The Trail-Making Test

[69], [70] is a measure of cognitive flexibility and psychomotor function. The task has been widely used in the neuropsychological literature [71] and has been demonstrated to be sensitive to impairment caused by a range of phenomena including traumatic brain injury, exposure to toxic substances, concussion, drug and alcohol intoxication, emotional disturbance, dementia and cognitive slowing due to normal ageing [72].

The reasons for choosing these particular tests were as follows: Firstly, as mentioned above, each of these tests measure cognitive processes that have previously been shown to be sensitive to the effects of alcohol. Second, each of the tasks have been widely employed in the literature, are well-known and easily accessible. Third, these tests are suitable for repeated measures designs in that they are either free of potentials strategies that lead to abrupt changes in outcome, or can be easily altered to achieve this (i.e., see the Method section for details regarding changes to the TMT which were designed to remove any potential learning effects). Fourth, participants require no special prior knowledge to perform the tasks, and are able to perform the tasks with a minimum of instructions. Finally, each of the tasks can be completed in a short period of time (<10 minutes) ensuring that the battery can be completed within a time-period that minimizes any variability associated with rising and falling BACs.

The aims of this study were twofold: first, to compare the dose-related effects of alcohol across a wide range of key cognitive processes, and second, to compare the relative sensitivity of the different measures as indices of impairment caused by acute alcohol administration.

## Methods

### Participants

Ethical approval was obtained from the Royal Adelaide Hospital Research Ethics Committee, and written consent was obtained from each of the participants. Participants were drawn from the wider community via advertising placed in local newspapers and community billboards. Each participant’s eligibility was subject to the following criteria: (i) aged 18–45 years, (ii) not currently pregnant or lactating, (iii) no major medical or psychiatric conditions, (iv) no visual disorders, (v) no dependence on any substance (excluding nicotine), (vi) not taking medication having a stimulative or sedative action, and (vii) had consumed at least five alcoholic beverages on at least one occasion in the past month. The age range was chosen to ensure that the participants were old enough to be of legal drinking age, but young enough to ensure that they were unlikely to be affected by any deleterious effects of ageing upon cognitive abilities. Criterion vii was chosen to ensure that the participants had had prior experience with ingesting and functioning under the dose sizes of alcohol employed in the experiment. While we did not seek to distinguish between ‘light’ and ‘heavy’ drinkers, we felt it was important that the participants were all ‘experienced’ drinkers.

Participants (*N* = 56; 32 males and 24 females) were randomly assigned to either the experimental or control condition (*N* = 28 for both groups), subject to the gender split being equal across both conditions. Participants were asked for written consent prior to taking part in the study, and were paid $200 AUS on completion of testing.

### Cognitive Abilities Tests

*Brief Intellectual Ability scales.* This measure comprises three tests from the Woodcock-Johnson III [73]: Verbal Comprehension (crystallized ability); Concept Formation (fluid ability); and Visual Matching (perceptual speed). It takes approximately 15–20 min to complete. The BIA was administered once only on the first day of testing in order to gauge each participant’s level of intellectual ability.

As indicated in the introduction the repeated-measures test battery was comprised of the UFOV, SOPT, IT, TSP, SART and TMT. In the following we provide details of the specific programs, sub-tests, or materials used in our experiment. For the UFOV, IT and SART we employed the computerized versions of the task described in [74], [75] and [66], respectively. It should be noted that we employed sub-test three of the UFOV in our study.

For the TSP and SOPT we generated computerized versions of the tasks using MatLab. For the TSP we used 70-node stimuli taken from Dry et al [50], which also describes the task in detail. For the SOPT [62] we presented participants with 10–12- and 16-design versions, with each version repeated three times per test-run.

Finally, for the TMT [72] it was necessary to generate versions of forms –A and –B that were suitable for multiple presentations. In order to achieve this we employed the original stimuli at baseline, and vertical and/or horizontal symmetrically transformed versions of the original stimuli at the three subsequent presentations. The order of presentation was identical for all participants, and at each test point they first solved the TMT-A, followed by TMT-B.

### Procedure

Participants attended on two consecutive days. Day 1 was a familiarization session, with alcohol manipulation applied on the second day. A time-line for the experimental protocol is provided in Table 1. On day 1 the participants were screened in regards to the exclusion criteria and the presence of drugs or alcohol (urine and breath samples) and completed the Brief Intellectual Abilities scales. They were then introduced to the experimental tasks for the purposes of familiarization and practice. At each test-run (on both day1 and day2) participants completed the test battery in the following order: UFOV, SOPT, IT, TSP, SART and TMT. Completing this battery typically required 40–50 minutes. The test battery was completed three times on day 1, each time beginning approximately at the start of the hour. On day 2 the participants were again screened for drug and alcohol use. They completed a fourth practice session, following which experimental data were collected at four time-points: baseline, +60 minutes, +120 minutes and +180 minutes. At 15 minutes before each of the post-baseline time-points participants in the alcohol group were provided with a dose of vodka (37.5% alcohol v/v) mixed with 200 ml of sugar-free orange juice. The Widmark equation [76] was employed to calculate the volume of alcohol required to raise each participant’s BAC to 0.10% over three equal-sized doses (resulting in target values of 0.048%, 0.082% and 0.10% at +60, +120 and +180 minutes, respectively). The target BAC values of 0.048% and 0.082% were chosen as they correspond closely to the legal limits for driving in countries such as Australia and Canada (0.05%), or New Zealand and the United States (0.08%). The upper-limit target value of 0.10% was chosen as numerous previous studies have demonstrated reliable effects of cognitive impairment at this level of intoxication [2], [3], [4], [5], [6], [7], [8], [9].

**Table 1:** Timeline for tasks across days 1 and 2, and the target blood alcohol concentration (BAC) for the alcohol group participants on day 2.

Time	Day 1 Tasks	Day 2	
		Tasks	Alcohol group target BAC
9∶00 am	Screening, etc.	Screening	0.000
10∶00	↓	Practice 4	↓
11∶00		**Baseline testing**	**0.000**
12∶00	Practice 1	**+60 testing**	**0.048**
1∶00 pm	Practice 2	**+120 testing**	**0.082**
2∶00	Practice 3	**+180 testing**	**0.100**

The volume of dose (*D*) consumed across the three time-points was calculated as 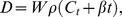 where *W* was body weight in kg, 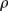 was the volume of distribution of alcohol in the body (L/kg), 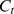 the BAC (g/100 ml) at time *t*, 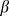 the elimination rate, and *t* the time (hours) from dose. Following Gullberg and Jones [77] we set 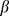 to 0.015 g/100 ml/hr and following Friels, Baer and Logan [78] we set 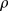 -values to 0.71 and 0.65 for males and females, respectively. The control group participants were provided with an equivalent volume of unsweetened orange juice.

Participants were told at the beginning of day 2 whether they were in the alcohol or control condition, hence the orange juice drunk by the control group participants was not a placebo. Although we are aware of the potential for expectancy effects we believe that: first, given that the participants were all experienced drinkers, and second, were aware of the relatively high projected BAC level of the alcohol condition group, that it was unnecessary and impractical to attempt to fool the control group participants into believing that they were drinking alcohol [79].

The participants were given 5 minutes to drink the dose and those in the alcohol condition thoroughly rinsed their mouths and throats with water to remove residual alcohol. BAC readings were collected from participants in the alcohol group prior to each of the six tests in the battery, using a Lion Alcometer 500 to chart the rise and fall of BAC levels across the test session.

## Results

### Participants

Mean age of participants in the alcohol (*M* = 26.42 years, *SD* = 6.19) and control (*M* = 26.63, *SD* = 7.79) conditions were markedly similar (*t* [54] = 0.11, *p* = .91) and groups were well matched on average for both the Brief Intellectual Abilities scales (*M = *108.57, *SD* = 11.16 and *M* = 106.71, *SD* = 12.75, for the alcohol and control groups, respectively: *t* [54] = 0.56, *p* = .57).

### Blood Alcohol Concentration

**Figure 1:**
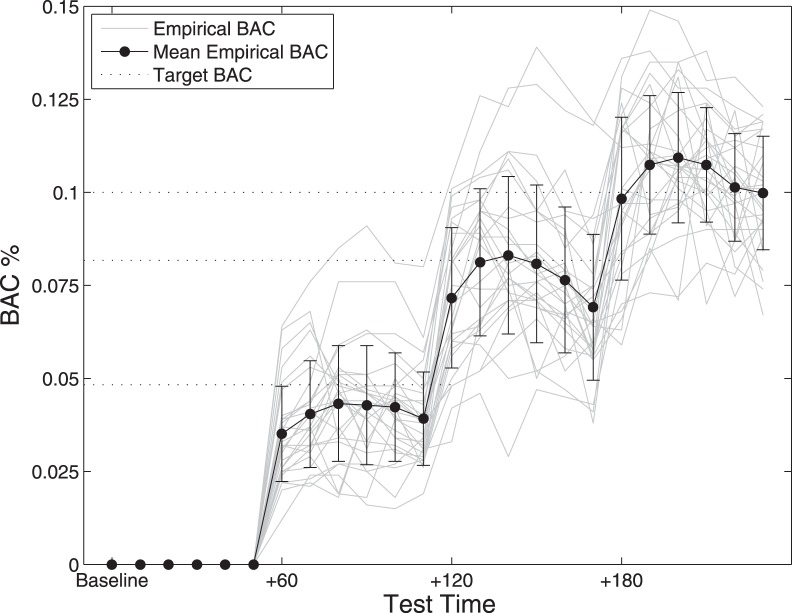
Means and error bars (±1 SD) for blood alcohol concentration (BAC), together with individual BAC measures and target BAC levels at baseline and three alcohol time points (0.048, 0.082 and 0.100%). The six data-points within each test block indicate the BAC reading at the commencement of each sub-test (i.e., Useful Field Of View, Self Ordered Pointing Task, Inspection Time, Traveling Salesperson Problem, Sustained Attention to Response Task and Trail Making Task).

Figure 1 shows the empirical BAC of participants in the alcohol condition. The Widmark equation-based prediction provided a close average correspondence between the target and observed BACs across each of the test blocks, with BACs of the majority well within 1 SD of the mean.

### Cognitive Abilities Tasks

Intercorrelations between the eight dependent variables from the six tests measured at baseline were small. Both measures from SART correlated weakly (*r* = .33, *p*<0.05), consistent with both aspects depending on sustained attention and there was low communality (*r* = .45, *p*<0.01) between TSP and TMT-A, consistent with both involving attentional monitoring and switching. Otherwise correlations were statistically nonsignificant and, taken together, these results suggested that the various tasks were measuring different aspects of cognitive functioning.

### Effects of Alcohol

During preliminary training all tasks showed improvement across the four practice sessions, with performance tending towards asymptotic by the final practice session (data not presented here). There were also qualitative differences between the performance levels of the two groups across the practice and baseline test-runs. Given widespread acknowledgement of the inappropriateness of expressing pretest/postest effects as difference-from-baseline scores [80], [81] it was necessary to correct for these differences at baseline level. First, for each task the shared variance between each participant’s baseline score and their score at each subsequent experimental test point (i.e., +60 min, +120 and +180) was partialled out using linear regression. This process ensured that each of the participants (and therefore both of the groups) were equivalent on all tasks at baseline.

Second, data for each of the dependent variables were transformed to z-scores, which were calculated across the pooled data of the experimental and control conditions and all four test-points. This allows for easy comparison across each of the different cognitive abilities tasks. The following analyses used baseline-normalized data.

**Figure 2:**
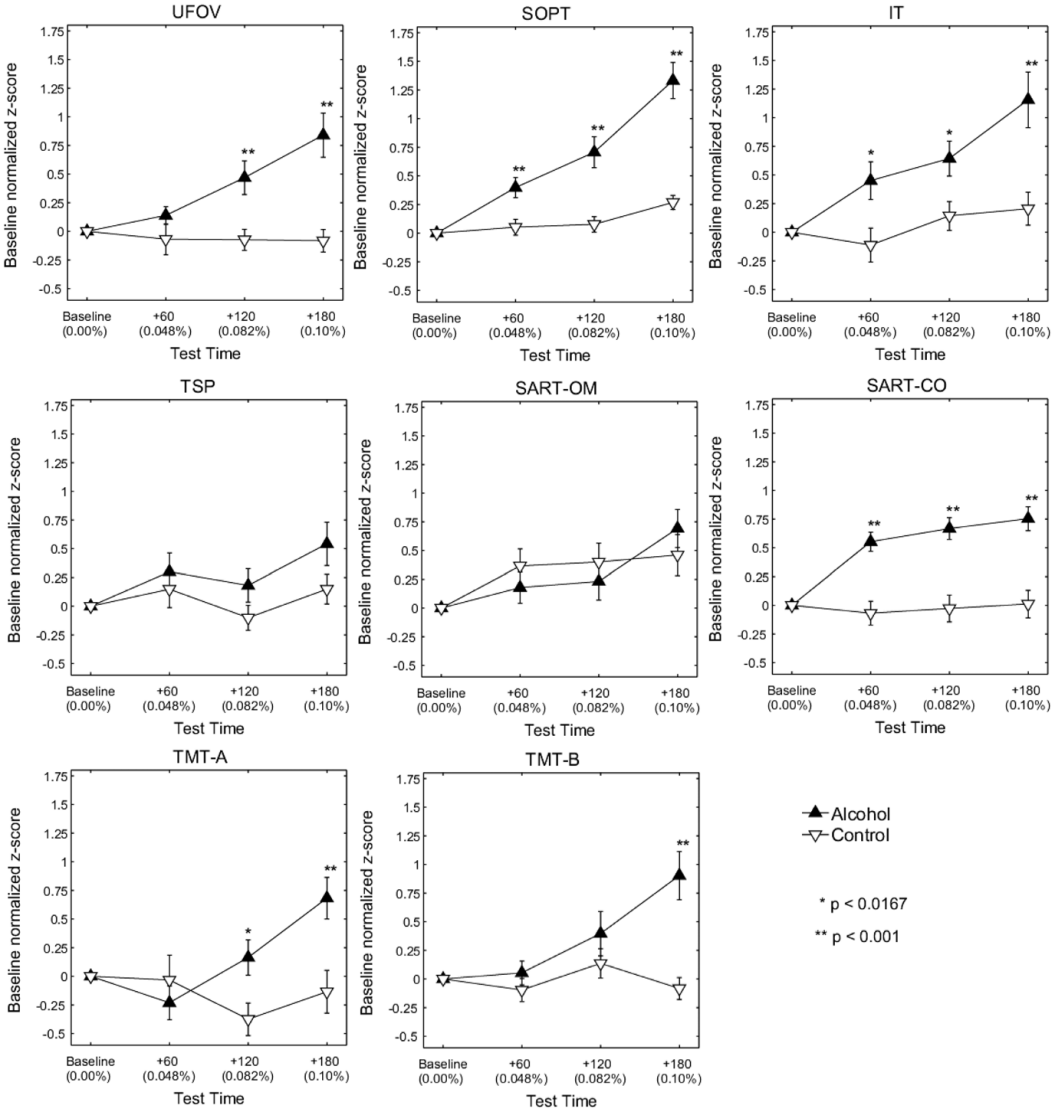
Comparison of the mean baseline-normalized performance of the alcohol and control group participants across the eight dependent variables from the six cognitive abilities tests. Error bars indicate one standard error of the mean. Significant differences between the control and alcohol groups are indicated by asterisks. UFOV = Useful Field of View; SOPT = Self-Ordered Pointing Task; IT = Inspection Time; TSP = Travelling Salesperson Problem; SART-OM = Sustained Attention Response Task – errors of omission; SART-CM = Sustained Attention Response Task – errors of commission; TMT-A = Trail Making Test A; TMT-B = Trail Making Test B.

Figure 2 compares the mean performance of the alcohol and control group participants across the six cognitive abilities tasks. Bonferroni adjustment for the number of tests has been applied because of multiple comparisons (*α = *0.05/3 = 0.0167). For the majority of the tests there was a clear qualitative difference in the performance of the control and experimental groups with highly statistically significant differences at the highest blood alcohol concentration. Repeated measures analysis of variance indicated that for each of the tasks excepting TSP and the SART-OM there was a strong and significant main effect of alcohol, with effect sizes (partial 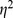) ranging from 0.07 to 0.37 (Table 2).

**Table 2:** F-tests for the main effect of alcohol, associated post-hoc comparisons, and effect sizes for each of the dependent variables associated with the six cognitive abilities tasks.

							Post-Hoc Comparisons (Alcohol vs. Control)								
	Main effect of alcohol						+60 (BAC ≈ 0.048%)			+120 (BAC ≈ 0.082%)			+180 (BAC ≈ 0.100%)		
	*F* [1], [54]		*p*		*η* ^2^		*t* [54]	*p*	*d*	*t* [54]	*p*	*d*	*t* [54]	*p*	*d*
UFOV	**18.37**		**0.00**		**0.25**		1.33	0.18	0.36	**3.13**	**0.00**	**0.78**	**4.27**	**0.00**	**1.00**
SOPT	**30.85**		**0.00**		**0.36**		**3.09**	**0.00**	**0.76**	**4.17**	**0.00**	**0.97**	**6.26**	**0.00**	**1.28**
IT	**13.98**		**0.00**		**0.20**		**2.54**	**0.01**	**0.65**	**2.53**	**0.01**	**0.65**	**3.37**	**0.00**	**0.83**
TSP	2.55		0.11		0.04		0.65	0.51	0.18	1.54	0.12	0.41	1.72	0.09	0.45
SART - OM	0.04		0.83		0.00		–0.92	0.36	–0.25	–0.72	0.47	–0.19	0.94	0.34	0.25
SART - CM	**32.13**		**0.00**		**0.37**		**4.64**	**0.00**	**1.06**	**4.57**	**0.00**	**1.05**	**4.63**	**0.00**	**1.06**
TMT – A	**4.33**		**0.04**		**0.07**		–0.75	0.45	–0.20	**2.52**	**0.01**	**0.66**	**3.14**	**0.00**	**0.78**
TMT – B	**8.28**		**0.00**		**0.13**		1.03	0.30	0.28	1.12	0.26	0.30	**4.27**	**0.00**	**1.00**

Note: *F*-tests with *p*<0.05 and *t*-tests with *p*<0.0167 are indicated in bold. Negative *d*-values indicate an advantage in the direction of the alcohol condition.

However, the data also provide strong evidence that the tasks varied in their sensitivity to the effects of alcohol. For only three tasks (SOPT, IT and SART-CO) was there a significant effect at the lowest blood alcohol concentration of 0.048%. Of these, the SOPT and IT showed a strong dose effect, with a clear relation between blood alcohol concentration and degree of impairment, whereas SART-CO performance deteriorated markedly at BAC 0.048% but changed little thereafter across BAC range 0.048% to 0.10%.

Given the variability in BACs across the participants at each of the test-time points (Figure 1), we felt it was important to re-run the analyses controlling for both baseline performance and the individual BAC of alcohol group participants at each of the dosed time-points. Importantly, this analysis indicated no difference in the basic pattern of results. Similarly, analyses employing the participants’ BIA and STW scores as covariates made no difference to the basic pattern of results.

## Discussion

These results have shown that impairment due to alcohol intoxication is not uniform across different domains of cognitive processing. Test results were relatively independent across the different tasks at baseline, suggesting that the test battery covered a range of relatively independent cognitive processes. Furthermore, comparing the performances of the control and alcohol groups across time-points +60 min to +180 min demonstrated that the different cognitive processes tapped by the different tasks were affected differentially by rising BACs. Thus, both the size of the dose effect and the magnitude of the change in effect across different dose levels were quantitatively different for different cognitive processes. These results have potential to inform our understanding of cognitive functioning in general; tests of working memory and attention as applied in this study have sometimes been regarded as reflecting a common executive function but current results cast doubt on the utility of executive processing as a distinct construct because the different measures have been shown to change in quantitatively different ways in response to a single experimental manipulation.

All variables except TSP (complex spatial problem solving) and SART-OM (vigilance for frequently occurring events) showed effects of alcohol intoxication; but only SOPT (working memory), IT (processing speed) and SART-CO (vigilance for and inhibition of response to rare events) showed significant deterioration at BAC 0.048%. The failure of TSP and SART-OM to show any significant effects was unexpected, although other researchers have reported null results at BAC 0.048% [82] and for BACs between 0.059 to 0.067 [83], for similarly complex tasks involving higher order cognitive processes. Clearly, why some tasks involving multiple-step, goal-directed activities may be relatively resistant to higher levels of intoxication is a question warranting further investigation.

The second aim of this study was to assess the suitability of these widely applied tests of cognitive functioning to provide a single marker for alcohol induced impairment that was sensitive to dose-related effects. Arguably, SART-CO has failed the criterion of providing a marker test for dose-related alcohol effects because almost all of the increase to error rate was registered at BAC 0.048% and thereafter errors did not increase much further. This test appeared to be particularly sensitive to disinhibition, although there also appeared to be a ceiling effect, with insufficient trials on which a response should be withheld. Results from the UFOV task showed similar trends to those observed for SOPT and IT and the UFOV is a very convenient test, taking only about 5 min to complete. However, although conceived primarily as a test of processing speed, the procedure followed also requires visual search and divided attention and there is therefore uncertainty about what processes are being measured. Moreover, decline in performance in the alcohol group at BAC 0.048 was not statistically significantly different from control performance, an outcome that mirrored results reported by Puell and Barrio [22], who tested participants at BACs around 0.03 to 0.05% but found no effect on UFOV performance.

On the other hand, both SOPT and IT were clearly sensitive in a dose-related way, to a similar extent and both tap fundamental processes that must underpin virtually all cognitive functioning: working memory and processing speed, respectively. However, SOPT is arguably a much less convenient task than IT. IT can be estimated in as little as 5 min, whereas SOPT requires at least 10 min and frequently 15 min to complete. Previous studies have employed shorter versions of the SOPT, for example Peterson et al [84] employed a 12 card version of the task with three repeats, and Pihl et al [28] a single presentation of 12 card problem. However, in both cases the test failed to distinguish between sober and intoxicated individuals even at BACs as high as 0.10%, presumably because the task was too limited in regards to both relative complexity (number of cards needed to be memorized) and number of repetitions. Furthermore, conceptually IT is a much simpler task; the judgement required is trivially easy given sufficient target exposure duration, and IT has been successfully measured in people with very low IQ, children as young as 5 years old and elderly people in excess of 90 years of age [85].

The outcome for IT at BAC 0.048% is similar to that reported by Tzambazis and Stough [15] for BACs around 0.05%, but our study demonstrates that performance on this task continues to decline as a function of rising BAC level. Importantly, considerable background research has confirmed that IT monitors a low level process that is heritable, underpins higher level general intelligence, is sensitive to both normal and less successful functional ageing and, unlike IQ, is stable across generations [85], [86]. Moreover, the measure has suitable task properties for wide-spread application; it is theoretically sound, highly reliable, non-invasive and convenient to use and there is now a sufficiently large accumulation of relatively recent measures with the exact procedure followed here to permit the generation of norms across the life span [2518 participants: 1504 males, aged from 6 to 92 years; 87]. We suggest, therefore, that IT has strong potential as a single test, sensitive to the time course of alcohol intoxication. Furthermore, given the established legal limits set for alcohol intoxication in regards to activities such as driving, we would anticipate that IT provides a potential method for comparing the effects of other sedative drugs like methadone, benzodiazepine and tetrahydrocannabinol.

Finally, the results of the current experiment suggest two obvious follow-up experiments. Firstly, given the large effect sizes for SOPT, IT and the SART tasks at BACs of 0.048% (d = 0.76, 0.65 and 1.06, respectively) it would be surprising if these tasks were not significantly sensitive to impairment at BAC levels <0.05%. Secondly, numerous studies have demonstrated that alcohol induced cognitive impairment differs qualitatively and quantitatively across the rising and falling limbs of BAC curves due to the phenomenon of acute tolerance 9]. In the current study we tested impairment for increasing BACs only - it would be of obvious interest to determine if the various tests employed in this study (and IT in particular) are similarly sensitive to impairment on the post-peak limb of the BAC curve. We are currently planning experiments to explore these two issues.
